# Identification and Functional Characterization of a Novel De Novo *SATB1* Frameshift Variant in a Patient with Epilepsy-Dominant Neurodevelopmental Disorders

**DOI:** 10.3390/genes17050565

**Published:** 2026-05-15

**Authors:** Mingchao Xu, Rui Zhang, Shiqi Fan, Miao Sun, Xue Zhang

**Affiliations:** 1 McKusick-Zhang Center for Genetic Medicine, Institute of Basic Medical Sciences & School of Basic Medicine, Chinese Academy of Medical Sciences & Peking Union Medical College, No.5 Dongdan Santiao, Dongcheng District, Beijing 100005, China; 18940048482@163.com (M.X.);; 2 State Key Laboratory for Complex Severe and Rare Diseases, Institute of Basic Medical Sciences & School of Basic Medicine, Chinese Academy of Medical Sciences & Peking Union Medical College, No.5 Dongdan Santiao, Dongcheng District, Beijing 100005, China

**Keywords:** *SATB1*, neurodevelopmental disorders, epilepsy, de novo variant, frameshift variant, nonsense-mediated mRNA decay (NMD), truncated protein, genotype-phenotype correlation

## Abstract

Background/Objectives: As a global chromatin organizer, SATB1 is increasingly implicated in neurodevelopmental disorders (NDDs). This study aims to delineate the clinical and molecular characteristics of a novel de novo *SATB1* variant in a patient presenting with epilepsy-dominant NDDs phenotypes. Methods: Triggered by the onset of seizures, trio-based whole-exome sequencing (Trio-WES) was performed to identify the genetic etiology. Subsequent sleep electroencephalogram (EEG) and magnetic resonance imaging (MRI) were then conducted to further characterize the patient’s clinical phenotypes. Pathogenicity was assessed through structural modeling and functional characterization. Nonsense-mediated mRNA decay (NMD) status, protein expression profiles, and subcellular localization were determined by reverse-transcription quantitative PCR (RT-qPCR), Western blotting, and immunofluorescence staining. The transcriptional regulatory impacts of the variant were quantified using dual-luciferase reporter system targeting known downstream regulatory elements. Clinical responses to antiepileptic intervention was also monitored. Results: We identified a novel de novo heterozygous pathogenic frameshift variant in *SATB1* (NM_002971.5: c.1718_1719insCA; p.Val574Argfs*134) in a patient presenting with early-onset epilepsy, mild intellectual developmental disorder (IDD), speech delay, and dental anomalies. Functional assays demonstrated that the variant-derived transcript escaping NMD, yielding a truncated protein that forms irregular punctate aggregates within nuclei. Dual-luciferase assays revealed significantly increased transcriptional activity, indicating a loss of the protein’s innate transcriptional regulatory capacity. Clinically, treatment with sodium valproate (VPA) successfully stabilized seizures of the patient, markedly reducing both frequency and intensity. Conclusions: The study reports a novel *SATB1* frameshift variant that exerts pathogenicity significant functional impairment by disrupting protein localization and transcriptional regulation. These findings expand the genetic spectrum of *SATB1*-related NDDs and underscore the efficacy of targeted antiepileptic management in genetic diseases.

## 1. Introduction

Neurodevelopmental disorders (NDDs) encompass a diverse group of conditions originating during the early development, typically characterized by persistent deficits in physical, cognitive, and social functioning [1]. The widespread implementation of genomic sequencing technologies has established genetic factors as primary contributors to NDDs pathogenesis [2]. Specifically, pathogenic variants in chromatin-modifying genes have emerged as key drivers, as they disrupt global transcriptional networks through extensive epigenetic dysregulation [3]. Notably, recent investigations have identified various deleterious *SATB1* variants as significant contributing factors to the etiology of NDDs [4].

Special AT-rich sequence-binding protein 1 (SATB1) functions as a chromatin organizer that scaffolds nuclear architecture through interactions with matrix attachment regions (MARs), dynamically coordinating gene expression via three-dimensional chromatin restructuring [5]. Beyond its well-established roles in regulating T-cell development [6], emerging evidence underscores the requirement of SATB1 for orchestrating transcriptional programs during neurogenesis and brain development [7]. Currently, *SATB1* is associated with two autosomal dominant syndromes in the Online Mendelian Inheritance in Man (OMIM) database: Developmental delay with dysmorphic facies and dental anomalies (DEFDA, MIM #619228) and Den Hoed–de Boer–Voisin syndrome (DHDBV, MIM #619229) [8]. These conditions share clinical features, including developmental delay, speech delay, and facial dysmorphisms. To date, 13 protein-truncating and 18 missense variants in *SATB1* have been documented (Figure S1).

In this study, we report a patient with early-onset epilepsy, mild intellectual developmental disorder (IDD), speech delay, and dental anomalies, harboring a novel de novo heterozygous frameshift variant in *SATB1*. Through clinical evaluation and functional characterization, we demonstrate the pathogenicity of the variant and its disruptive impact on the SATB1 protein function. Our findings not only expand the known genotypic and phenotypic landscapes of *SATB1*-related NDDs but also provide evidence-based insights into the pharmacological management of epilepsy in this patient population.

## 2. Materials and Methods

### 2.1. Ethic Approval

Clinical information of the patient and blood samples of the family trio were collected and the informed consent was obtained. This study was approved by Peking Union College Institutional Review Board in accordance with the Declaration of Helsinki.

### 2.2. Trio-WES and Sanger Sequencing

Trio-based whole-exome sequencing (Trio-WES) was performed on the patient and both parents. Genomic DNA was extracted using QIAamp DNA Blood Mini Kit (QIAGEN, Hilden, Germany; Cat. No. 51106). Sequencing was performed using the Illumina Novaseq 6000 system, obtaining a mean coverage of targeted regions of 99.78% at 100 × read depth, with high sensitivity and specificity in variant detection. DNA libraries were sequenced using Novaseq 6000 platform (Illumina, San Diego, CA, USA). Sequence reads were then mapped to the reference human genome (GRCh38/hg38) using Burrows-Wheeler Aligner (BWA) software (v.0.7.17) [9]. Alignment files were sorted and duplicated reads were marked using Sambamba (v.0.6.8) [10], after which the processed files were utilized to generate sequencing statistics, including coverage and read depth. Functional annotation was performed using ANNOVAR (v. 2023Feb1) [11].

Candidate variants were prioritized through a structured multi-step filtering pipeline: (1) Population frequency filtering: Variants were retained with a minor allele frequency (MAF) < 1% in both global and East Asian populations in databases including Genome Aggregation Database (gnomAD) (v.3.1.2). (2) Technical quality control: To ensure high sequencing quality, a minimum allele balance (AB) of 0.2 was required. (3) Functional impact assessment and clinical evidence review: Nonsynonymous, frameshift, and splicing variants were prioritized and further assessed for pathogenicity using in silico prediction tools, including MutationTaster. Variants documented as “Benign” in the ClinVar database were excluded to focus on clinically relevant findings. (4) Phenotype-driven prioritization: Clinical manifestations of the patient were standardized into Human Phenotype Ontology (HPO) terms. The Exomiser (v.13.1.0) [12] was then utilized to rank variants, specifically focusing on those associated with neurodevelopment-related phenotypes.

The corresponding sequences of primers for Sanger sequencing are provided in Table S1.

### 2.3. Three-Dimensional Protein Modeling, Visualization and Pathogenicity Evaluation

The SATB1 protein sequence was downloaded from UniProt and aligned. Structural models of wild-type (WT) and variant SATB1 proteins were generated using Alphafold (v.3) [13]. Key structural domains and functional regions were separated and visualized using PyMOL (v.3.1.1) [14].

The pathogenicity of the detected variant was evaluated according to the American College of Medical Genetics and Genomics/Association for Molecular Patholoogy (ACMG/AMP) standard and guidelines for the interpretation of sequence variants [15].

### 2.4. RNA Extraction and RT-qPCR

Total RNA was extracted from peripheral blood samples using Trizol LS reagent (Invitrogen, Carlsbad, CA, USA; Cat. No. 10296010CN). 1 μg of purified RNA was reverse-transcribed into complementary DNA (cDNA) using PrimeScript II Reverse Transcriptase (Takara Bio, Kusatsu, Japan; Cat. No. 2690) under standard reaction conditions. Quantitative PCR (qPCR) was performed with Hieff qPCR SYBR Green Master Mix (Low Rox) (Yeasen Biotechnology, Shanghai, China; Cat. No. 11202ES03). *SATB1* expression levels were normalized to *ACTB*.

### 2.5. SATB1 Expression Constructs Generation, Cell Culture and DNA Transfection

WT *SATB1* and corresponding variant sequences were cloned into a pEYFP-C1 vector respectively. All constructs were confirmed by Sanger sequencing. HEK293T cells (Cell Resource Center, Institute of Basic Medical Sciences, CAMS/PUMC) were cultured in Dulbecco’s modified Eagle Medium (DMEM) (Cell Resource Center, Institute of Basic Medical Sciences, CAMS/PUMC) supplemented with 10% fetal bovine serum (FBS) (Invitrogen; Cat. No. 10091148), 1% penicillin-streptomycin (Cell Resource Center, Institute of Basic Medical Sciences, CAMS/PUMC), and 1% GlutaMAX (Invitrogen; Cat. No. 35050061) at 37 °C with 5% CO_2_. Cells were transfected at 70~80% confluence with relatively equal amounts of DNA using Lipofectamine 3000 (Invitrogen; Cat. No. L3000015) in Opti-MEM (Invitrogen; Cat. No. 31985070) according to the manufacturer’s instructions.

### 2.6. Extraction of Protein and Western Blotting

Cells were washed with phosphate buffered saline (PBS) (Gibco, Grand Island, NY, USA; Cat. No. 10010023) and lysed in radioimmunoprecipitation assay (RIPA) lysis buffer (Beyotime Biotechnology, Shanghai, China; Cat. No. P0013C) supplemented with protease and phosphatase inhibitor cocktails (Roche Diagnostics, Basel, Switzerland; Cat. No. 05892970001; Roche Diagnostics; Cat. No. 04906837001). Protein concentrations were determined using BCA Protein Assay Kit (Solarbio Life Sciences, Beijing, China; Cat. No. PC0020). Equal amount of protein (30 μg) were heated at 95 °C for 10 min.

The protein was separated with TGX Stain-Free FastCast Acrylamide Kit 10% (Bio-rad Laboratories, Hercules, CA, USA; Cat. No. 1610183), and transferred onto Immobilon-PSQ PVDF Membrane (Merck, Darmstadt, Germany; Cat. No. ISEQ00010). After blocking with Tris-buffered saline-Tween 20 (TBST) buffer (Solarbio Life Sciences; Cat. No. T1085) containing 5% skim milk (Solarbio Life Sciences; Cat. No. D8340), membranes were then incubated with primary antibodies that diluted in primary antibody dilution buffer (Beyotime Biotechnology; Cat. No. P0023A): rabbit anti-GFP antibody (Cell Signaling Technology, Danvers, MA, USA; Cat. No. 2956, 1:2000) and mouse anti-β-actin antibody (Cell Signaling Technology; Cat. No. 3700, 1:2000). Following incubation with HRP conjugated secondary antibodies (Cell Signaling Technology; Cat. No. 7074; Cell Signaling Technology; Cat. No. 7076) that diluted in TBST, protein bands were visualized with ECL substrate (Tanon Science & Technology, Shanghai, China; Cat. No. 180-5001).

### 2.7. Dual-Luciferase Reporter Assays

The *IL-2* promotor and *IgH*-MAR core element were separately cloned into pGL3-basic vector. The target sequence of the former encompasses the promoter region of human *IL-2* gene, while the latter’s target sequence consists of seven repeats of the 5′-TCTTTAATTTCTAATATATTTAGAAttc-3′ MAR sequence [10,11,12]. HEK293T cells were co-transfected with reporter plasmid, the pRL-TK Renilla luciferase control, and either WT or variant SATB1 expression construct. Luciferase activity was measured 48 h post-transfection using Dual-Luciferase Reporter Assay System (Promega, Madison, WI, USA; Cat. No. E1960). Firefly luciferase activity was normalized to Renilla activity.

### 2.8. Statistical Analyses

Statistical analyses were conducted using Graphpad Prism (v.10). Statistical significance was assessed using Fisher’s exact tests. Data in dual-luciferase assays were analyzed by one-way ANOVA tests. *p*-value < 0.05 was considered statistically significant.

## 3. Results

### 3.1. Case Presentation

**Figure 1:**
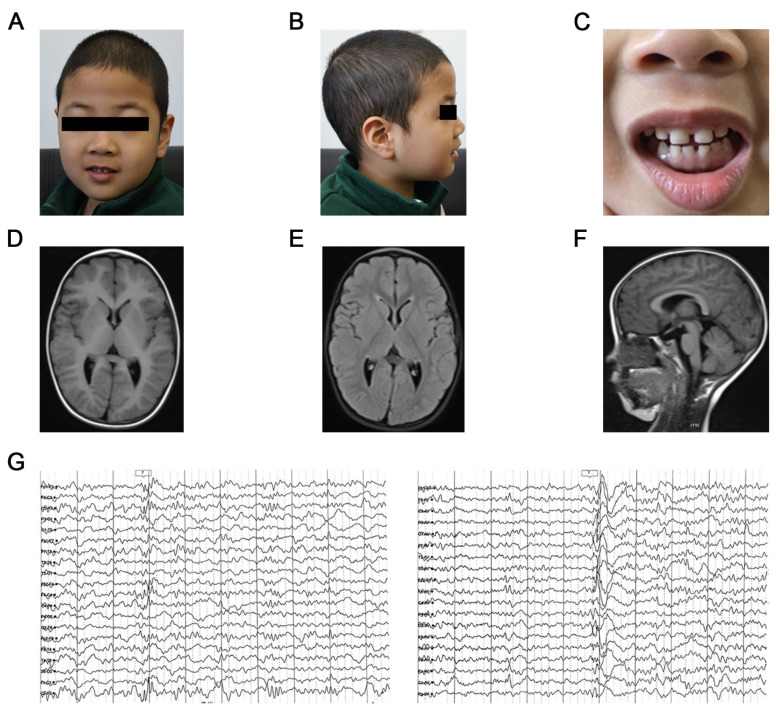
Representative clinical profiles of the patient. (**A**,**B**) Frontal and lateral facial photographs of the patient; (**C**) Frontal view of the patient’s teeth; (**D**–**F**) Brain MRI at age 2: (**D**) axial T1-weighted, (**E**) axial T2-FLAIR, and (**F**) Sagittal T1-weighted scans; (**G**) Representative sleep EEG recordings obtained at age 5 following VPA treatment.

The patient was at the age of two and a half years with his first convulsions. His healthy non-consanguineous parents exhibited no remarkable clinical phenotypes and had no family history of NDDs or other congenital anomalies. Genetic testing identified the de novo variant in *SATB1*. Subsequent to the diagnosis of IDD, the patient presented with his initial epileptic seizure. After a cluster of four nocturnal seizures, comprehensive evaluation including brain magnetic resonance imaging (MRI) and sleep electroencephalogram (EEG) was performed at age 3. While brain MRI revealed preserved brain architecture and normal parenchymal signals (Figure 1D–F; Table 1), the detection of myoclonic and generalized tonic-clonic seizures (GTCS) activities on EEG confirmed the diagnosis of epilepsy. On gross clinical examination, no significant craniofacial dysmorphisms were observed (Figure 1A,B; Table 1); the patient’s physical growth was within normal limits. However, the patient manifested dental anomalies, specifically characterized by delayed tooth eruption and increased interdental spacing (Figure 1C; Table 1).

Given that early seizure control is critical for mitigating neurological impairment and facilitating functional restoration in NDDs [16,17], pharmacological intervention with sodium valproate (VPA) was initiated at an initial dosage of 20mg/kg·d. Following two brief recurrences within two weeks, the VPA dosage was titrated up to 40mg/kg·d gradually. The measured serum VPA concentrations (53 μg/mL and 68 μg/mL) during this period confirmed that the drug levels were approaching the targeted therapeutic range (50~100 μg/mL) [18].

Over the ensuing two years (as of March 2025), the patient reveived a maintenance dosage of VPA at 40 mg/kg·d and experienced no further seizures. Routine therapeutic drug monitoring (TDM) performed every six months showed that serum VPA concentrations remained stable and within therapeutic range. Clinical monitoring showed a marked resolution of EEG abnormalities by age 5 (Figure 1G).

### 3.2. Identification and In Silico Analysis of SATB1 Variant

**Figure 2:**
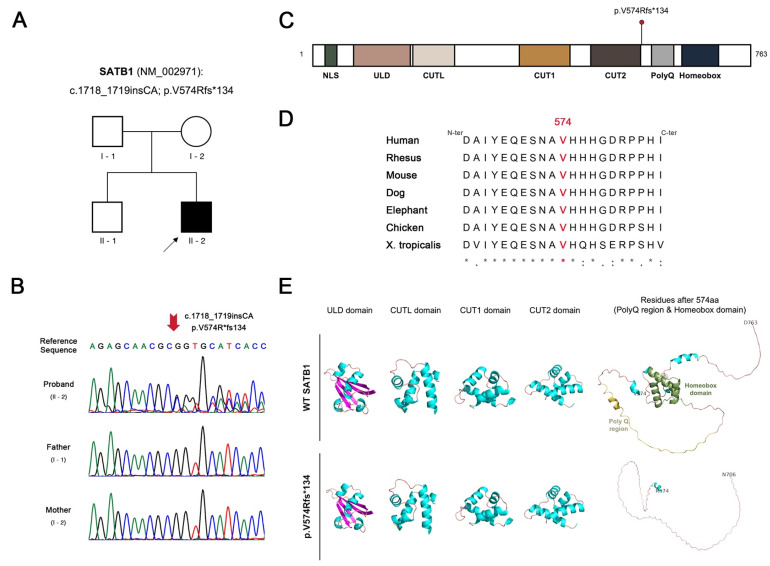
Genetic identification and structural characterization of SATB1 p.Val574Argfs*134 variant. (**A**) Pedigree of the family, with the patient indicated by an arrowhead; (**B**) Sanger sequencing chromatograms of the family trio; (**C**) Schematic representation of the SATB1 (NP_002962) protein domains, with the location of the SATB1 p.Val574Argfs*134 variant marked by a red dot; (**D**) Multiple sequence alignment of residue 574 (highlighted in red) across diverse species. Asterisks (*) indicate positions with a fully conserved residue; (**E**) Predicted three-dimensional domain structures of WT and variant SATB1 proteins. Structural architectures are color-coded: α-helices (cyan), β-sheets (magenta), loops (salmon), PolyQ region (yellow-orange), and the Homeobox domain (smudge).

Analysis of Trio-WES data revealed a heterozygous *SATB1* variant (NM_002971.5: c.1718_1719insCA; p.Val574Argfs*134) in the patient, consistent with an autosomal dominant inheritance pattern (Figure 2A). Sanger sequencing confirmed the de novo origin of the variant, as it was absent in both parents (Figure 2B). As of April 2026, the variant has not been documented in major clinical variant databases including Human Gene Mutation Database (HGMD) and Clinvar. The residue V574 and its adjacent regions exhibit high evolutionary conservation across multiple species (Figure 2D), suggesting a high pathogenic potential of the variant. The resulting frameshift at residue 574 introduces a premature stop codon, which is predicted to truncate several highly conserved functional domains (Figure 2C,  Figures S1 and S2). Protein structural modeling further reveals that the alteration entirely abolishes the polyglutamine (PolyQ) tract and the critical Homeobox domain, while the secondary conformation of the upstream functional regions remains intact (Figure 2E).

**Figure 3:**
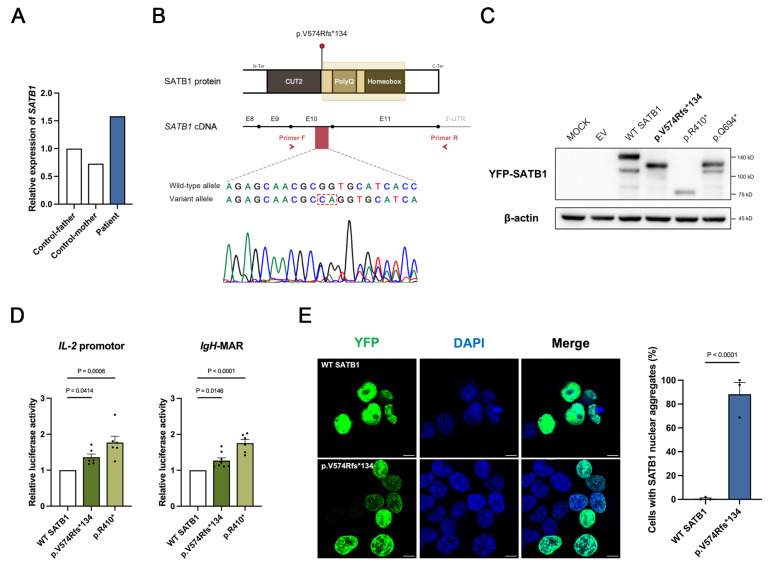
Molecular and functional characterization of SATB1 p.Val574Argfs*134 variant. (**A**) qPCR analysis verification of NMD escape in patient-derived peripheral blood samples; (**B**) The relative location of SATB1 p.Val574Argfs*134 variant and sequencing chromatogram of patient-derived cDNA. (**C**) Immunoblot analysis of SATB1 protein expression in HEK293T cells. SATB1 p.Arg410* and SATB1 p.Gln694* served as known NMD-escaping controls, respectively. β-actin was used as loading control. In the frameshift variant p.Val574Argfs*134, the asterisk denotes the position of the premature stop codon. In the nonsense variants p.Arg410* and p.Gln694*, the asterisks represent the termination codon. (**D**) Transcriptional regulatory activity of WT and variant SATB1 on downstream targets. *p*-values are indicated above the columns; (**E**) Representative immunofluorescence images displaying the subcellular YFP-SATB1 fusion proteins (green). Nuclei were counterstained with DAPI (blue). Scale bar = 10 μm. The right panel is statistical analysis of cells with SATB1 nuclear aggregates. *p*-values are indicated above the columns.

In accordance with the ACMG guidelines (ACMG SF v3.3) [19], no clinically significant secondary findings were identified in the WES data of the patient. The identified variant was classified as Pathogenic based on the following integrated evidence according to the ACMG/AMP guidelines [15]. First, PVS1 was fulfilled as the variant is a frameshift indel in *SATB1* where loss-of-function (LoF) is a well-established mechanism [4]; the variant removes more than 10% of the transcript (Figure 3C and Figure S2), thereby truncating a region critical to protein function. Second, PS2 was applied because of the confirmed de novo origin of the variant. Finally, PM2 was satisfied as the variant is absent in gnomAD.

### 3.3. Molecular Characterization of the SATB1 p.Val574Argfs*134 Variant

#### 3.3.1. Stable Expression of SATB1

To determine whether the frameshift variant triggers nonsense-mediated mRNA decay (NMD), we first assessed *SATB1* expression in peripheral blood of the patient. RT-qPCR analysis of the patient’s peripheral blood showed no significant reduction in total SATB1 transcript levels (Figure 3A). To exclude the possibility of compensatory expression from the WT allele, we performed Sanger sequencing of patient-derived cDNA. The resulting chromatogram revealed comparable signal intensities between the WT allele and mutant allele, indicating that the mutant transcript is stably expressed and escapes NMD (Figure 3B). Furthermore, Western blotting was employed to evaluate the protein expression profiles. Similar to the previously validated NMD-escaping variants (p.Arg410* and p.Gln694*) [4], the p.Val574Argfs*134 variant yielded a truncated protein (Figure 3C). Collectively, these findings suggest that the SATB1 p.Val574Argfs*134 variant escapes NMD.

#### 3.3.2. Impaired Transcriptional Regulatory Activity of the Variant Protein

To evaluate the impact of the variant on SATB1-mediated transcriptional regulation, we performed dual-luciferase reporter assays using two established downstream targets: the *IL-2* promoter and *IgH*-MAR [20,21,22]. Compared with the WT protein, the variant SATB1 exhibited significantly reduced trans-repressive activity on both target sequences (Figure 3D), indicating that the C-terminal truncation significantly impairs the protein’s normal regulatory capacity.

#### 3.3.3. Altered Subcellular Distribution of the Variant Protein

SATB1 is a nuclear matrix-associated protein that exerts its regulatory functions through chromatin interaction. To investigate whether the variant affects its spatial organization, we overexpressed YFP-tagged *SATB1* in HEK293T cells. Immunofluorescence analysis revealed that while WT SATB1 was evenly distributed within the nuclei, the variant protein formed conspicuous irregular punctate aggregates (Figure 3E). This distinct shift in spatial organization suggests that the variant disrupts the normal nuclear distribution of SATB1. Together, these molecular findings—characterized by NMD escape, compromised transcriptional regulatory activity, and aberrant subcellular localization—support the classification of SATB1 p.Val574Argfs*134 as a pathogenic variant.

## 4. Discussion

NDDs are a group of conditions originating during the developmental period, characterized by significant deficits that impair the attainment of cognitive, emotional, and motor milestones. Exploring the monogenic causes of NDDs not only provides crucial insights into the molecular mechanisms of neurodevelopment but also highlights potential therapeutic targets. The *SATB1* gene, encoding a pivotal chromatin organizer and transcriptional regulator, has emerged as a key player in neurodevelopment. Pathogenic variants in this gene are linked to a spectrum of NDDs characterized by IDD, epilepsy, and dysmorphic features [4,23,24]. In this study, we report a novel de novo heterozygous *SATB1* insertion variant (NM_002971.5: c.1718_1719insCA; p.Val574Argfs*134) in a patient presenting with epilepsy, mild IDD, speech delay, and dental anomalies.

VPA has been widely recognized as a first-line treatment for generalized epilepsy [25]. While a previously reported patient with a similar frameshift variant (SATB1: p.Leu710Valfs*42) required polytherapy (VPA and oxcarbazepine) for seizure control [26], patient in this study remained seizure-free after receiving a maintenance dosage of VPA alone, with serum VPA levels within the safe therapeutic range. Crucially, it must be acknowledged that these findings represent a clinical observation rather than a targeted therapy based on SATB1 mechanisms. Given the diverse genetic backgrounds and the inherent complexity of SATB1 function, the management may serve as a therapeutic reference for similar cases but cannot be generalized without further validation through large-scale cohort studies and mechanistic investigations.

To investigate the potential for NMD, we first quantified *SATB1* mRNA in the patient’s peripheral blood. RT-qPCR results indicated preserved mRNA stability. Notably, a slight upregulation was observed, potentially reflecting a compensatory transcriptional feedback mechanism to maintain homeostasis despite impaired SATB1 function. Using total *SATB1* mRNA to infer NMD status may conceal the possible absence of variant allele. To further support the conclusion that the variant escapes NMD, we performed Sanger sequencing on cDNA derived from the patient. The similar expression levels of both WT and variant alleles suggests that the variant escapes NMD. Dual-luciferase reporter assays using established SATB1 targets—the *IL-2* promoter and *IgH*-MAR [21]—confirmed that the variant protein exhibits significantly reduced trans-repressive activity. Mechanistically, this impairment is likely driven by subcellular spatial disorganization, as the variant protein mislocalizes into irregular punctate aggregates. These findings align with structural predictions of Homeobox domain disruption, a known pathogenic hotspot essential for DNA binding and chromatin remodeling [4].

Both p.Arg410* and p.Gln694* are nonsense variants of SATB1 that have been experimentally confirmed to escape NMD [4]. The p.Arg410* variant results in the loss of critical functional domains, significantly impaired transcriptional repression, and reduced chromatin binding affinity. Western blotting analysis further revealed diminished levels of the truncated protein, supporting haploinsufficiency (LoF) as its primary pathogenic mechanism. Conversely, the p.Gln694* variant also exhibits attenuated transcriptional repression (suggestive of LoF) but is uniquely characterized by the formation of abnormal nuclear aggregates. While such mislocalization suggests a potential dominant-negative effect through spatial interference within the nuclei, the clinical phenotypes of patients harboring these truncating variants are notably milder than those with gain-of-function (GoF) missense mutations of SATB1. This clinical evidence implies that the pathogenicity of NMD-escaping truncating variants stems predominantly from functional deficiency rather than potent proteotoxicity. Our identified p.Val574Argfs*134 variant mirrors the molecular characteristics of p.Gln694*, including its C-terminal location, NMD escape, reduced repressive activity, and formation of nuclear aggregates. Given the similarly mild clinical phenotypes in our patient, p.Val574Argfs*134 most likely exerts its pathogenic effect through LoF mechanism. Among SATB1 variants associated with NDDs, different types of variants often lead to clinical phenotypes of varying severity. This divergence highlights the importance of variant-specific analyses in deciphering the heterogeneous clinical manifestations of SATB1-related NDDs.

We acknowledge that the HEK293T cell model has limitations in imitating the complex context of central nervous system. Furthermore, as the cDNA-based expression constructs lack introns, they fail to facilitate the formation of the exon junction complex (EJC) during splicing; consequently, assessing NMD escape solely via western blotting in vitro is insufficient. While our current results provide evidence that the p.Val574Argfs*134 variant alters SATB1 behavior, future studies utilizing patient-derived induced pluripotent stem cells (iPSCs)-induced neurons or brain organoids will be essential to further elucidate its impact on neurodevelopment.

In summary, this study provides the first documentation of SATB1 p.Val574Argfs*134 frameshift variant as a pathogenic driver of NDDs. By integrating clinical observation with mechanistic validation, our findings expand the genotypic and phenotypic spectrum of *SATB1*-related NDDs, offering valuable evidence to support molecular diagnosis and precise genetic counseling for affected families.

## Figures and Tables

**Table 1:** Clinical features of the patient.

Clinical Features	Abnormalities in the Patient
Neurologic features	
Intellectual developmental disorder (HP:0001249)	Yes (mild)
Speech delay (HP:0031435)	Yes
Epilepsy (HP:0001250)	Yes
Hypotonia (HP:0001252)	No
Ataxia (HP:0001251)	No
Motor regression (HP:0033044)	No
brain MRI abnormalities	No
sleep EEG abnormalities	Yes
Other phenotypic features	
Facial dysmorphism (HP:0001999)	No
Dental anomalies (HP:0000164)	Yes
Hearing abnormality (HP:0000364)	No
Abnormality of vision (HP:0000504)	No
Skin abnormality (HP:0000951)	No
Hair abnormality (HP:0001595)	No
Abnormal nail morphology (HP:0001597)	No
Other abnormalities	No

## Data Availability

The data presented in this study are available on request from the corresponding author.

## Supplementary Materials

The following supporting information can be downloaded at: https://www.mdpi.com/article/10.3390/genes17050565/s1, Figure S1: Schematic representation of the SATB1 protein structure and reported pathogenic variants related to NDDs; Figure S2: Alignment between WT and variant SATB1 amino acid sequences; Table S1: Primer sequences for Sanger sequencing validation; Table S2: Primer sequences for qPCR analysis. Reference [27] is cited in the Supplementary Materials.

## Abbreviations

The following abbreviations are used in this manuscript:

NDD	neurodevelopmental disorder
Trio-WES	trio-based whole-exome sequencing
EEG	electroencephalogram
MRI	magnetic resonance imaging
NMD	nonsense-mediated mRNA decay
RT-qPCR	reverse-transcription quantitative PCR
IDD	intellectual developmental disorder
VPA	sodium valproate
SATB1	special AT-rich sequence-binding protein 1
MAR	matrix attachment region
OMIM	Online Mendelian Inheritance in Man
DEFDA	Developmental delay with dysmorphic facies and dental anomalies
DHDBV	Den Hoed-de Boer-Voisin syndrome
BWA	Burrows-Wheeler Aligner
MAF	minor allele frequency
gnomAD	Genome Aggregation Database
AB	allele banlance
HPO	Human Phenotype Ontology
WT	wild-type
ACMG/AMP	American College of Medical Genetics and Genomics/Association for Molecular Pathology
cDNA	complementary DNA
qPCR	quantitative PCR
DMEM	Dulbecco’s modified Eagle Medium
FBS	fetal bovine serum
PBS	phosphate buffered saline
RIPA	radioimmunoprecipitation assay
TBST	Tris-buffered saline-Tween 20
GTCS	generalized tonic-clonic seizures
TDM	therapeutic frug monitoring
HGMD	Human Gene Mutation Database
PolyQ	polyglutamine
LoF	loss-of-function
GoF	gain-of-function
EJC	exon junction complex
iPSC	induced pluripotent stem cell
