# Supplementary material for: Identification and Functional Characterization of a Novel De Novo SATB1 Frameshift Variant in a Patient with Epilepsy-Dominant Neurodevelopmental Disorders
Source: Genes (Basel). 2026 May 15;17(5):565. doi: 10.3390/genes17050565 (PMC13205537; doi:10.3390/genes17050565)
Supplement: Supplementary file 1 [file genes-17-00565-s001.zip › Supplementary Materials.pdf]

Supplementary Materials

**Figure S1**	-	Schematic representation of the SATB1 protein structure and reported pathogenic variants related to NDDs.
**Figure S2**	-	Alignment between WT and variant SATB1 amino acid sequences.
**Table S1**	-	Primer sequences for Sanger sequencing validation.
**Table S2**	-	Primer sequences for qPCR analysis.

Figure S1. Schematic representation of the SATB1 protein structure and reported pathogenic variants related to NDDs.

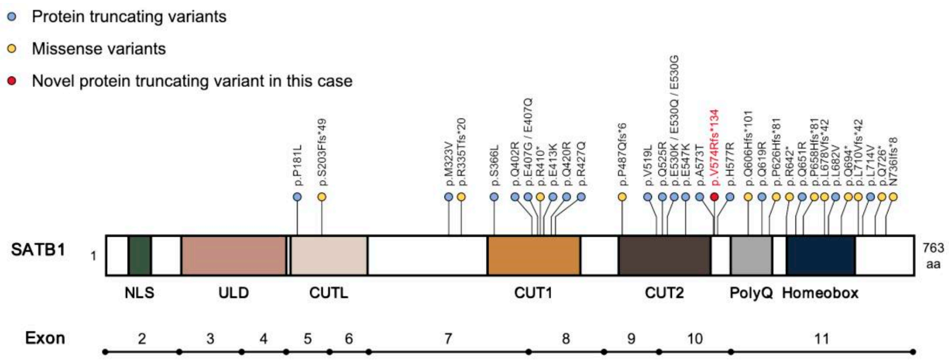

Functional domains are delineated by colored boxes, with previously reported pathogenic mutations superimposed at their respective positions [4,26,27]. These variants were curated from HGMD and peer-reviewed literature (as of April 2026).

Figure S2. Alignment between WT and variant SATB1 amino acid sequences.

Amino acid sequences of SATB1 (NP_002962) in normal situation

1	MDHLNEATQG	KEHSEMSNNV	SDPKGPPAKI	ARLEQNGSPL	GRGRLGSTGA	KMQGVPLKHS
61	GHLMKTNLRK	GTMLPVFCVV	EHYENAIEYD	CKEEHAEFVL	VRKDMLFNQL	IEMALLSLGY
121	SHSSAAQAKG	LIQVGKWNPV	PLSYVTDAPD	ATVADMLQDV	YHVVTLKIQL	HSCPKLEDLP
181	PEQWSHTTVR	NALKDLLKDM	NQSSLAKECP	LSQSMISSIV	NSTYYANVSA	AKCQEFGRWY
241	KHFKKTKDMM	VEMDSLSELS	QQGANHVNFG	QQPVPGNTAE	QPPSPAQLSH	GSQPSVRTPL
301	PNLHPGLVST	PISPQLVNQQ	LVMAQLLNQQ	YAVNRLLAQQ	SLNQQYLNHP	PPVSRSMNKP
361	LEQQVSTNTE	VSSEIYQWVR	DELKRAGISQ	AVFARVAFNR	TQGLLSEILR	KEEDPKTASQ
421	SLLVNLRAMQ	NFLQLPEAER	DRIYQDERER	SLNAASAMGP	APLISTPPSR	PPQVKTATIA
481	TERNGKPENN	TMNINASIYD	EIQQEMKRAK	VSQALFAKVA	ATKSQGWLCE	LLRWKEDPSP
541	ENRTLWENLS	MIRRFLSLPQ	PERDAIYEQE	SNA**VHHHGDR**	**PPHIIHVPAE**	**QIQQQQQQQ**
601	**QQQQQQQAPP**	**PPQPQQQPQT**	**GPRLPPRQPT**	**VASPAESDEE**	**NRQKTRPRTK**	**ISVEALGILQ**
661	**SFIQDVGLYP**	**DEEAIQTLSA**	**QLDLPKYTII**	**KFFQNQRYYL**	**KHHGKLKDNS**	**GLEVDVAEYK**
721	**EEELLKDLEE**	**SVQDKNTNTL**	**FSVKLEEELS**	**VEGNTDINTD**	**LKD***	

Amino acid sequences of SATB1 (NP_002962) in patient
Chr3:g.18352052_1835053insTG; c.1718_1719insCA; p.V574Rfs*134

1	MDHLNEATQG	KEHSEMSNNV	SDPKGPPAKI	ARLEQNGSPL	GRGRLGSTGA	KMQGVPLKHS
61	GHLMKTNLRK	GTMLPVFCVV	EHYENAIEYD	CKEEHAEFVL	VRKDMLFNQL	IEMALLSLGY
121	SHSSAAQAKG	LIQVGKWNPV	PLSYVTDAPD	ATVADMLQDV	YHVVTLKIQL	HSCPKLEDLP
181	PEQWSHTTVR	NALKDLLKDM	NQSSLAKECP	LSQSMISSIV	NSTYYANVSA	AKCQEFGRWY
241	KHFKKTKDMM	VEMDSLSELS	QQGANHVNFG	QQPVPGNTAE	QPPSPAQLSH	GSQPSVRTPL
301	PNLHPGLVST	PISPQLVNQQ	LVMAQLLNQQ	YAVNRLLAQQ	SLNQQYLNHP	PPVSRSMNKP
361	LEQQVSTNTE	VSSEIYQWVR	DELKRAGISQ	AVFARVAFNR	TQGLLSEILR	KEEDPKTASQ
421	SLLVNLRAMQ	NFLQLPEAER	DRIYQDERER	SLNAASAMGP	APLISTPPSR	PPQVKTATIA
481	TERNGKPENN	TMNINASIYD	EIQQEMKRAK	VSQALFAKVA	ATKSQGWLCE	LLRWKEDPSP
541	ENRTLWENLS	MIRRFLSLPQ	PERDAIYEQE	SNA**RCITMAT**	**GRPTLSMFQQ**	**SRFSNSSSNS**
601	NSSSSSSRHR	RLHSHSSSHR	QALGSPHGNP	**RWPLQQSQMR**	**KTDRRPGHEQ**	**KFQWKPWESS**
661	**RVSYKTWACT**	**LTKRPSRLCL**	**PSSTFPSTPS**	**SSSFRTSGTI**	**SSTTAN***	

Residue numbering is annotated according to the corresponding biological isoform. Amino acid alterations are specifically denoted in red text to indicate the substitution sites.

Table S1. Primer sequences for Sanger sequencing validation.

SATB1-forward (Sanger)	CCAAAAGCCAGGGATGGTTG
SATB1-reverse (Sanger)	AGTGGCACTGTTGAACGAAAC

The sequences are listed in the 5' → 3' orientation.

Table S2. Primer sequences for qPCR analysis.

SATB1-forward	GCGATGAACTGAAACGAGCAGG
SATB1-reverse	CATAGCCCGAAGGTTTACCAGC
ACTB-forward	CACCATTGGCAATGAGCGGTTC
ACTB-reverse	AGGTCTTTGCGGATGTCCACGT

The sequences are listed in the 5' → 3' orientation.
